# Intricate relationship between cancer stemness, metastasis, and drug resistance

**DOI:** 10.1002/mco2.710

**Published:** 2024-09-21

**Authors:** Tikam Chand Dakal, Ravi Bhushan, Caiming Xu, Bhana Ram Gadi, Swaranjit Singh Cameotra, Vikas Yadav, Jarek Maciaczyk, Ingo G. H. Schmidt‐Wolf, Abhishek Kumar, Amit Sharma

**Affiliations:** ^1^ Genome and Computational Biology Lab Department of Biotechnology Mohanlal Sukhadia University Udaipur Rajasthan India; ^2^ Department of Zoology M.S. College Motihari Bihar India; ^3^ Department of General Surgery The First Affiliated Hospital of Dalian Medical University Dalian China; ^4^ Department of Molecular Diagnostics and Experimental Therapeutics, Beckman Research Institute City of Hope Monrovia California USA; ^5^ Stress Physiology and Molecular Biology Laboratory Department of Botany Jai Narain Vyas University Jodhpur Rajasthan India; ^6^ SAS Polyclinic Mohali Punjab India; ^7^ School of Life Sciences Jawaharlal Nehru University New Delhi India; ^8^ Department of Stereotactic and Functional Neurosurgery University Hospital of Bonn Bonn Germany; ^9^ Center for Integrated Oncology (CIO) Department of Integrated Oncology University Hospital Bonn Bonn Germany; ^10^ Manipal Academy of Higher Education Manipal Karnataka India; ^11^ Institute of Bioinformatics International Technology Park Bangalore India

**Keywords:** cancer stem cells, cancer stemness, drug resistance, EMT, metastasis, signaling pathways, tumor microenvironment

## Abstract

Cancer stem cells (CSCs) are widely acknowledged as the drivers of tumor initiation, epithelial‐mesenchymal transition (EMT) progression, and metastasis. Originating from both hematologic and solid malignancies, CSCs exhibit quiescence, pluripotency, and self‐renewal akin to normal stem cells, thus orchestrating tumor heterogeneity and growth. Through a dynamic interplay with the tumor microenvironment (TME) and intricate signaling cascades, CSCs undergo transitions from differentiated cancer cells, culminating in therapy resistance and disease recurrence. This review undertakes an in‐depth analysis of the multifaceted mechanisms underlying cancer stemness and CSC‐mediated resistance to therapy. Intrinsic factors encompassing the TME, hypoxic conditions, and oxidative stress, alongside extrinsic processes such as drug efflux mechanisms, collectively contribute to therapeutic resistance. An exploration into key signaling pathways, including JAK/STAT, WNT, NOTCH, and HEDGEHOG, sheds light on their pivotal roles in sustaining CSCs phenotypes. Insights gleaned from preclinical and clinical studies hold promise in refining drug discovery efforts and optimizing therapeutic interventions, especially chimeric antigen receptor (CAR)‐T cell therapy, cytokine‐induced killer (CIK) cell therapy, natural killer (NK) cell‐mediated CSC‐targeting and others. Ultimately use of cell sorting and single cell sequencing approaches for elucidating the fundamental characteristics and resistance mechanisms inherent in CSCs will enhance our comprehension of CSC and intratumor heterogeneity, which ultimately would inform about tailored and personalized interventions.

## Graphical abstract

Complexity of cancer stem cell biology and the intricate relationship between cancer stem cells, metastasis, and drug resistance

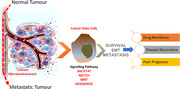

## 1 INTRODUCTION

Cancer is the increasing cause of death in the world.^1^, ^2^ While the prevalence of contagious diseases has dramatically decreased over the past years, leukemia and solid tumors have been demonstrated to have an increasing prevalence overall.^3^ Key elements contributing to the development of cancer include an increase in the average lifespan, the accumulation of genetic alterations, and a favorable microenvironment.^4^, ^5^, ^6^ The majority of treatments use potent cytotoxic chemicals to attack particular uncontrolled components in an effort to reduce tumor survival and cell growth.^7^ Cancer adapts to hostile surroundings and can endure therapeutic management due to its quick reproduction capability and continual mutations. The property of cancer cells to proliferate and, in many cases, survive is largely due to their stemness.^8^, ^9^, ^10^, ^11^, ^12^ Thus, developing more potent treatments may be made possible by understanding how cancer cells acquire and evolve resistance.

This review is motivated by the urgent need to confront the persistent challenge of cancer, with its profound impact on global health, by delving into the elusive realm of cancer stem cells (CSCs). Recognizing their pivotal role in therapy resistance and disease recurrence, we aim to unravel the intricate cellular (hypoxia, oxidative stress, and others), molecular and signaling pathways governing CSCs behavior, thus identifying novel targets for therapeutic intervention. Through this exploration, we advocate for a paradigm shift in cancer therapeutics toward targeted interventions tailored to CSCs vulnerabilities, emphasizing the importance of understanding CSCs biology and its influence on the tumor microenvironment (TME). CSCs have been proposed as the underlying cause of medication resistance and cancer recurrence, mostly attributed to their ability for self‐renewal and differentiation into several cancer cell.^13^ Contrary to the physiological function of adult SCs, cancer cells display all features of stemness, are unable to maintain tissue‐level and cellular homeostasis, and support the development of cancer.^14^ Being able to self‐renew and differentiate, as well as the construction of microenvironments that support stemness, are all stemness properties shared by SCs and cancer cells that serve as a base for cancer maintenance and survival.^4^, ^9^, ^15^, ^16^ As they may instruct nearby cells to produce nutrition and work together to elude the immune system, CSCs have amazing organizing abilities that help to foster the growth of tumors. CSCs produce heterogeneous cell populations that frequently have high plasticity.^10^, ^17^ and show increased resistance to stressors in the TME (like low O_2_ or nutritional status) or to the initiation of apoptosis by therapeutic drug agents,^11^, ^18^ and quiescence as a typical response.^12^, ^19^ Ultimately, our review serves as a rallying cry for concerted scientific efforts to decipher the complexities of CSC biology and pave the way for transformative advancements in personalized cancer treatment strategies.

A significant number of clinical trials predominantly assess the efficacy of medicines specifically targeting CSCs, either on their own or in conjunction with conventional treatments.^20^, ^21^ Nevertheless, the inconsistency in the outcomes gained emphasizes the necessity of enhancing the transfer of discoveries from preclinical investigations to their assessment in diseased individuals. Therefore, based on the current evidence, it is recommended that the most beneficial treatments consider the different types of cancer, the presence of specific CSC biomarkers, the use of advanced techniques that specifically target these markers, and the combination of these new treatments with the most effective standard treatments or conventional therapies.

**FIGURE 1:**
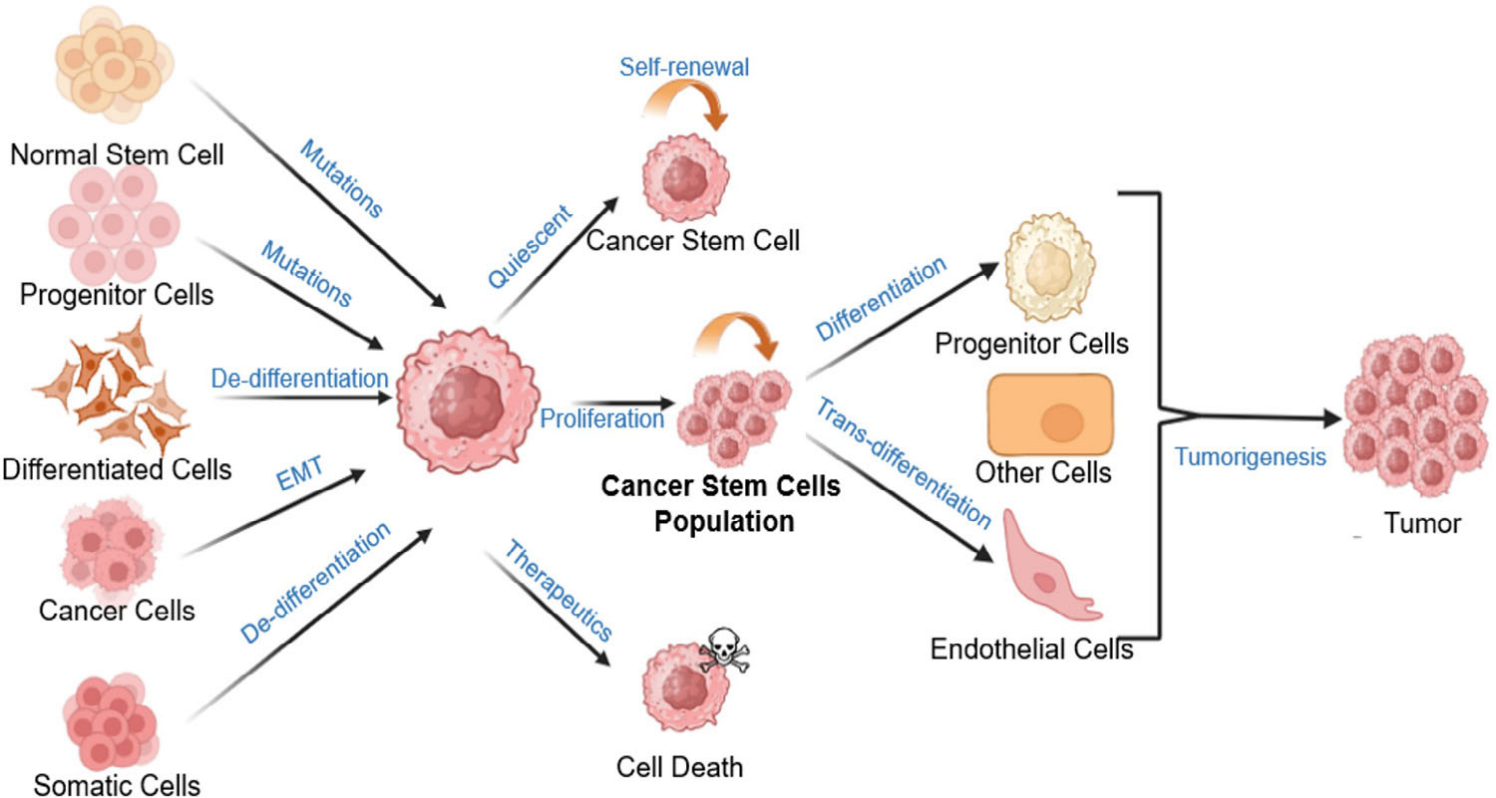
A detailed representation of cancer stem formation, differentiation, and role in tumorigensis. Figure has been drawn with the help of Biorender.

This review comprehensively discusses the intricate mechanisms driving CSCs behavior and their profound implications in therapy resistance and disease progression. Key focal points include elucidating the molecular and cellular pathways governing CSCs self‐renewal, differentiation, and interaction with the TME. Additionally, the review explores the role of CSCs in driving the epithelial–mesenchymal transition (EMT), modulating signaling cascades, and subverting apoptotic pathways, shedding light on their contributions to metastasis, therapeutic resistance, and disease relapse. Highlighting the urgent need for targeted interventions tailored to CSCs vulnerabilities, the review underscores the transformative potential of understanding CSC biology using preclinical and clinical studies in revolutionizing cancer treatment strategies. This review delves into the nexus of CSCs, metastasis, and drug resistance, illuminating their pivotal role in tumorigenesis and therapeutic evasion, pathways involved in cancer stemness as well as its pathophysiological implications. We delineate the functions of stem cells in the generation of TME associated at cancerous site (Figure 1). An insight into the distinct role of CSCs in promoting the EMT, the role of signaling pathways, and the degradation of apoptotic pathways and death receptors has been discussed (Figure 1). Given the dynamic cellular events that occur during cancer progression and the contributions of CSCs to resistant nature, understanding their molecular and cellular regulatory mechanisms in a heterogeneous nature is critical for the development of CSC‐targeted therapies. Beginning with a comparison of CSCs to normal stem cells, we dissect how CSCs drive tumor heterogeneity and survival. We then focus on key signaling pathways like JAK/STAT, WNT, NOTCH, and HEDGEHOG, elucidating their contributions to therapy resistance.

The aim of this review is to explore the intricate relationship between hypoxia, oxidative stress status, and TME with cancer stemness. Through an exploration of CSCs interactions with the TME, we unveil adaptive mechanisms fostering tumor progression. The review also seeks to elucidate the molecular and signaling pathways involved in cancer stemness and its pathophysiological implications, particularly focusing on the EMT and metastasis. The contributions of CSCs in disease progression and therapeutic drug resistance have also been discussed to shed light on their pivotal roles in tumorigenesis and therapeutic evasion. By highlighting major challenges and summarizing key clinical studies, we underscore the urgency of decoding CSC‐mediated resistance for targeted therapies, aiming to enhance patient outcomes in oncology. Finally, conducting a comprehensive discussion, the review aims to provide insights into the dynamic cellular events being orchestrated by CSCs in TME, emphasizing the urgent need to understand CSC biology for the development of targeted therapies and improved patient outcomes in oncology.

## 2 LINKING HYPOXIA AND CELLULAR OXIDATIVE STATUS TO CSCs

This subsection delves into the pivotal role of hypoxia and cellular oxidative status within the TME. Known as the “Cancer Stem Niche,” this milieu comprises fibroblasts, immune cells, and hypoxic regions, regulating CSCs self‐renewal and differentiation.^22^ Cancer‐associated fibroblasts (CAFs), which are present in the tumor stroma, are thought to be a primary cause of TME. Debate surrounds TME cell origin, with CAFs emerging as key regulators. CAFs are activated as a major component of the tumor stroma during carcinogenesis.^23^ CAFs secrete a variety of extracellular matrix (ECM) proteins, chemokines, cytokines, and growth factors, all of which contribute significantly to tumor progression, invasion, and metastasis.^23^, ^24^, ^25^ CAFs secrete factors promoting tumor progression, survival, and CSCs phenotype induction across various cancers. CAFs are also associated with poor survival in most cancers and are being investigated as potential therapeutic targets.^26^ CAF‐released factors enhance tumor cell survival by stimulating antiapoptotic pathways or triggering the EMT and the acquisition of CSC characteristics. This has been demonstrated in melanoma, non‐small cell lung cancer, and colorectal cancer.^27^, ^28^, ^29^, ^30^, ^31^, ^32^

Altogether, these elements regulate multiple self‐renewal pathways, which helps to keep the CSC population's stemness.^33^ The TME has been identified as a significant influencing element in the development of tumors. Although the most likely model is a hybrid of the above two techniques, the focus of this work is on CSCs. There are various thoughts on the origins of TME cells; the two most frequent are that these cells come from nearby tissues or that they develop from cancer cells.^34^, ^35^, ^36^ Vermeulen and coauthors and others.^36^, ^37^ revealed that fibroblasts restore the CSCs phenotype and retain the stemness trait of colon CSCs by activating the Wnt/β‐catenin signaling. Quail and Joyce's findings further proved that coinjection of stromal cells and cancer cells causes tumor formation in mice.^34^ According to this study, stromal cells in mice act as a favorable milieu for the growth of cancer cells.

Hypoxia plays a crucial role in the initiation and advancement of tumors. Hypoxia‐inducible factor (HIFs) serves as the critical regulator of transcription process under low oxygen levels. Hypoxia in solid tumors activates HIF, fostering cell proliferation, angiogenesis, and therapy resistance. It governs the expression of genes that respond to hypoxia, hence facilitating enhanced cell proliferation, survival, angiogenesis, invasion, metastasis, and resistance to therapeutic interventions. Tumor hypoxia links to EMT and CSCs, crucial for tumor maintenance. The process of EMT and the presence of CSCs have been found to be closely linked to tumor hypoxia, a condition characterized by low oxygen levels. ECM molecules, like laminin and hyaluronan, support CSCs, promoting tumorigenesis and resistance. HIF‐1 also induces EMT in clear cell renal carcinoma, aiding metastasis. CSCs exhibit reactive oxygen species (ROS) scavenger upregulation, resisting apoptosis and therapy. Strategies targeting CSCs and TME components, like CAFs, offer hope against therapy resistance and tumor progression. This association is significant as it influences the regulation and sustenance of the CSCs phenotype. Previous studies have provided evidence that the induction of EMT can be observed in several human cancer cell lines when exposed to in vitro conditions. However, EMT's complexity challenges therapeutic development. Understanding these interactions is vital for effective cancer treatment.

**FIGURE 2:**
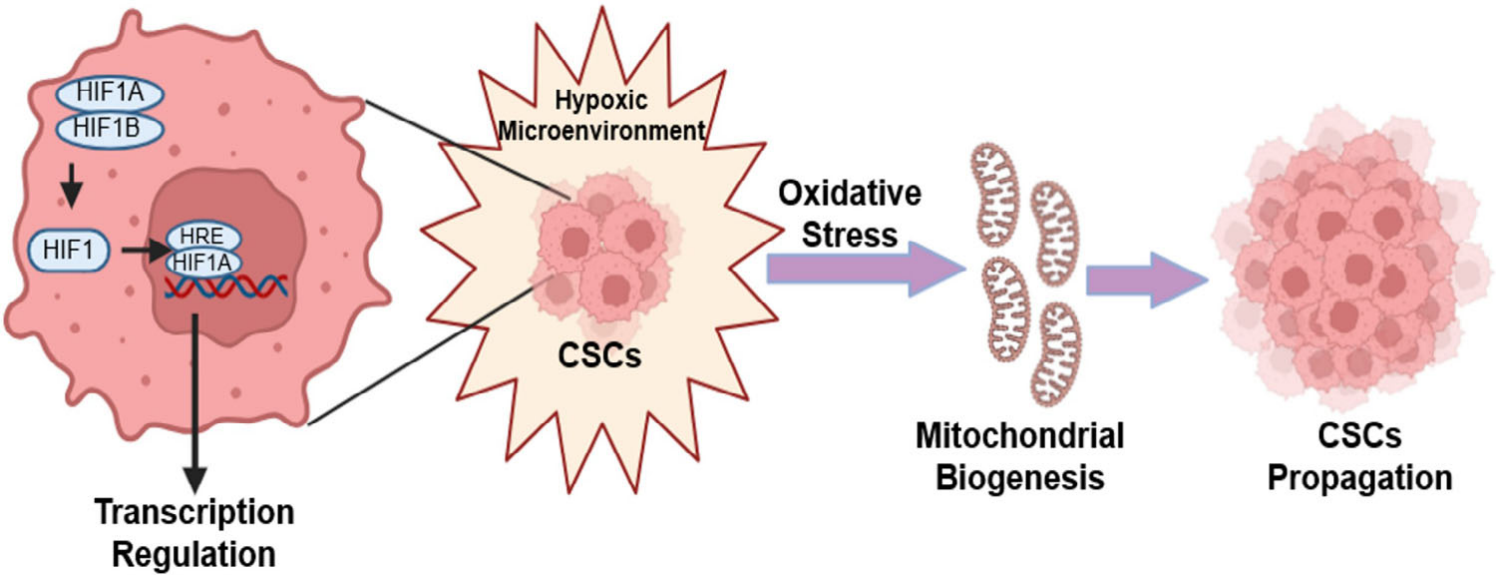
The figure illustrates the interplay between cancer stem cells (CSCs), hypoxic conditions, and cellular oxidative status within the tumor microenvironment. Figure has been drawn with the help of Biorender.

The TME's hypoxic character, which develops as a result of the cancer cells’ rapid development, is another salient trait. It is widely known that HIFs, which are activated in hypoxic situations, cause cancer.^38^ This phenomenon is believed to be primarily driven by the activation of HIF‐1, as suggested by the findings of Imai and coauthors, Lester and coauthors, and Yang and coauthors.^39^, ^40^, ^41^ HIFs activate a number of pathways that encourage CSCs proliferation, self‐renewal, and tumor formation when oxygen levels are low.^42^ Furthermore, it has been shown that the CSCs percentage in the TME increases in low‐oxygen environments^43^ (Figure 2). Furthermore, there have been reports suggesting that HIF‐1 has a role in facilitating EMT in clear cell renal cell carcinoma. This process entails the indirect inhibition of E‐cadherin by stimulating the production of ZEB1 and 2, as well as the E2A immunoglobulin enhancer‐binding proteins E12/E47. This leads to the acquisition of EMT attributes by the tumors.^44^, ^45^

CSCs exhibit diminished levels of ROS as a result of heightened expression of ROS scavengers, which serve to mitigate ROS‐mediated DNA damage and apoptosis.^46^, ^47^ Notably, the administration of the ROS scavenger N‐acetylcysteine effectively reinstated the characteristic traits of CSCs.^48^ The chemical known as Salinomycin has the ability to selectively eliminate CSCs. It achieves this by specifically targeting the CD44+CD24− subset of cells and increasing the levels of ROS.^49^ The reversal of NRF2 silencing resulted in the loss of CD44+ cells’ capacity to maintain increased status of ROS and their susceptibility to anticancer medications.^50^ The reduction of glutamine resulted in a drop in the fraction of SP cells due to an elevation in intracellular levels of ROS.^51^ Additionally, glycolysis facilitated the upregulation of doublecortin‐like kinase 1 expression and sustained the characteristics of CSCs by maintaining low levels of ROS in pancreatic cancerous cells resistant to gemcitabine.^48^ Nevertheless, a study conducted by Lee and coauthors demonstrated that the myelocytomatosis (*MYC*) and myeloid cell leukemia‐1 collaborate to sustain the resistant nature of CSCs to chemotherapy.^52^ This resistance is achieved by the augmentation of ROS production and HIF‐1α expression. This phenomenon can perhaps be attributed to the autonomy of the apoptotic domain. The blocking of HIF‐1α resulted in the inhibition of CSCs growth and the restoration of sensitivity to chemotherapy.

In addition to these, a number of other TME components cause EMT, metastasis, and chemoresistance. For instance, pancreatic cancer resistance to chemotherapy and CAFs radiation.^53^ Additionally, in some cancer models, coculturing CAFs together with cancer cells promotes tumor initiation, angiogenesis, invasion, and metastasis.^54^, ^55^ Together, eradicating CSCs and preventing tumor growth will be possible by targeting elements of the TME. These strategies’ clinical application is expected to reenergize the fight against a persistent obstacle. These results demonstrate the intricate involvement of multiple variables in promoting EMT in carcinoma cells. These findings pose a significant obstacle in the development of effective therapeutic strategies aimed at regulating EMT in tumor cells.^44^, ^45^

## 3 MOLECULAR MECHANISM UNDERLYING CANCER STEMNESS

Cancer progression drives through alterations in gene expression orchestrated by various signaling pathways, including MAPK/ERK, TGFβ‐SMAD, and WNT/β‐catenin and others. Besides this, EMT induction by stromal signals and oxidative stress leads to cellular phenotypic changes, including loss of polarity and enhanced invasiveness, reminiscent of physiological EMT programs. Elevated matrix metalloproteinase (MMP) levels facilitate ECM degradation, promoting tumor invasion. Moreover, EMT confers resistance to chemotherapy and evasion of immune surveillance. Understanding the complex interplay between EMT and CSCs offers insights into therapeutic resistance mechanisms and metastatic potential.

### 3.1 Role of signaling pathways

Multiple oncogenic signaling systems regulate the phenotypic and genotypic traits of CSCs. Multiple studies have shown that cancer cells gain stem‐like properties when there is inappropriate activation of normal stem cell development pathways such as Wnt, Notch, Hedgehog, and JAK–STAT signaling.^56^, ^57^ The function of these pathways in controlling CSCs has been demonstrated in many cancer models, such as pancreatic, colorectal, breast, glioma, nephroblastoma, osteosarcoma, melanoma, lung, and hepatocellular carcinoma.

Oncogenic signaling pathways like JAK/STAT, Wnt, Notch, and Hedgehog play pivotal roles in regulating CSCs across various cancer types. The regular functioning of these pathways, which are typically involved in the formation of tissues and maintaining their balance, is disrupted in cancer. This disruption causes tumor cells to acquire and retain traits associated with stem cells. Gaining a comprehensive understanding of the complex interaction between these signaling cascades and CSC biology is essential for the development of precise therapeutic approaches and the enhancement of clinical results.

#### 3.1.1 JAK/STAT signaling

Hematopoiesis, neurogenesis, and stem cell self‐renewal are all maintained by JAK/STAT signaling. Among all STAT protein subtypes, STAT3 is crucial for cellular survival and proliferation.^58^ The cytoplasmic domain of the receptor undergoes conformational changes as a result of the binding of numerous ligands, including cytokines/chemokines (such as interferons & interleukins), hormones, and growth factors, to their corresponding receptors. JAK proteins activate STAT3 by phosphorylating Tyr705. Phosphorylation by JAK in receptor cytoplasmic domains and by mTOR/MAPK at Ser727 further activates STAT3.^59^ The nuclear translocation of activated STAT3 controls the transcription of its targets, including c‐Myc, cyclin D1, and Bcl‐2.^60^

STAT3 plays a crucial role in signaling many cells’ developmental processes, such as stem cell maintenance and differentiation. STAT3 is essential for regulating both embryonic and adult stem cells in both mice and humans.^61^ The ability of mouse embryonic stem cells to renew themselves is controlled by the tyrosine phosphorylation of STAT3. Moreover, STAT3 engages with Notch ligands Delta‐like 1 and regulates the precursor neurons in the mouse neocortex in the initial stages of development.^62^

However, STAT3 is constitutively activated in diseases like cancer. Numerous human malignancies, including colorectal, breast, melanoma, prostate, ovarian, thyroid, glioblastoma, bone, hepatic, nephroblastoma, leukemia, and pancreatic CSCs, exhibit hyperactivation of STAT3.^63^, ^64^, ^65^, ^66^, ^67^ STAT3 orchestrates oncogenic pathways, crucial in tumorigenesis and cancer stemness. Acetylated STAT3, in complex with CD44 and p300, translocates to the nucleus, regulating target genes like Myc. It interacts with NF‐κB and HIF‐1, enhancing CD133+ subset.^68^ Breast cancer and prostate CSCs were discovered to have significantly activated JAK/STAT signaling and its mediators IFNK, IFNGR, IL6, and CSF2.^69^ Pancreatic CSCs are targeted via STAT3 inhibition. ALDH+ and CD44+/CD24+ subsets exhibit heightened p‐STAT3. Inhibiting STAT3 reduces tumorsphere formation and STAT3 target gene expression in these subsets.^65^ Additionally, stem cells from myelodysplastic syndrome and acute myeloid leukemia have been found to have aberrant STAT3 activation.^66^

STAT3 activation drives multipotency in glioma stem cells (GSCs). TGF‐β regulates GSC self‐renewal and differentiation via JAK/STAT pathway. Inhibiting STAT3 enhances radiotherapy effectiveness.^70^ Additionally, it was found that STAT3 suppression reduced the proliferation of tumor spheres and as well as the expression of the neural SCs genes Nestin and Olig2 in glioblastoma cells.^67^ JAK/STAT pathway regulates CSC‐driven metastasis. In colon CSCs from metastatic samples, IL‐11 levels correlate with TGF‐β and SMAD2 expression in stroma, leading to STAT3 activation in tumor cells.^68^ Collectively, these data point to the critical function of JAK/STAT signaling in controlling CSCs in numerous cancer models. The phenotype and genotype of CSCs have been found to be inhibited by a number of STAT3 inhibitors, demonstrating the CSCs regulatory role of STAT3.

#### 3.1.2 Wnt signaling

The Wnt signaling pathway is a evolutionarily conserved and functions both canonically (where β‐catenin is required) and noncanonically (where β‐catenin is not required).^71^ The function of Wnt signaling in tissue homeostasis and embryonic development has been thoroughly outlined.^71^, ^72^ The confirmation of the association between Wnt signaling and stem cells may be established as Wnt ligands are derived from cells that support stem cells.^73^ Experimental data point to the function of canonical Wnt signaling in controlling stem cells in the intestine, mammary glands, skin, and hair follicles.^72^ In adult mouse models, it has been demonstrated that β‐catenin mutations and the expression of the Wnt inhibitor Dkk1 hinder hair follicle growth and cause the intestinal epithelium to degrade.^74^ Additionally, it has been demonstrated in preclinical mouse models that the Wnt/β‐catenin downstream targets TCF and LGR5 have a significant impact on controlling intestine and skin stem cells.^75^ However, a variety of human malignant neoplasms, including skin, lung, colon, brain, liver, urinary tract, pancreatic, intestine, and leukemia, have been shown to exhibit dysregulation of Wnt signaling mediators like Axin, β‐catenin, APC, and Wnt1 for tumor initiation and maintenance.^76^, ^77^, ^78^, ^79^, ^80^

Canonical Wnt signaling is said to be very important in controlling CSCs.^81^, ^82^ In colorectal cancer precursor lesions, the relationship between Wnt signaling and CSCs has been demonstrated.^83^ In skin, lung, colon, brain, liver, urinary tract, pancreatic, intestine, and prostate cancer, Wnt/β‐catenin signaling upregulates the stemness of cancer cells by modulating CSC markers LGR5, CD24, Prom1, CD44, Oct‐4, and c‐Myc.^82^ Wnt signaling influences R‐spondin 2′s control over GSCs. GSCs exhibit elevated levels of R‐spondin, β‐catenin, c‐Myc, c‐Met, AXIN2, MMP9, MMP7, LGR4‐6, Nestin, and SOX2 compared with parental cells. Wnt high subpopulations of glioblastoma cells express higher levels of stemness markers, suggesting Wnt's role in maintaining stem‐like traits.^84^

#### 3.1.3 Notch signaling

In multicellular organisms, Notch signaling is a cellular developmental route. Cellular proliferation, cell fate determination, differentiation, cell communication, and embryonic development are all facilitated by this genetically conserved molecular pathway.^56^, ^57^, ^85^ Four Notch transmembrane receptors (Notch 1−4) and five membrane‐bound cell surface ligands (JAG 1 and 2, DLL 1, DLL 3, and DLL 4) are involved in mammalian Notch signaling.^56^, ^85^, ^86^ Notch receptors are fragmented by two‐step proteolytic process mediated by ADAM proteases (ADAM ‐ 10 or 17) and secretase throughout the course of ligand–receptor interaction, releasing the active cleaved portion Notch intracellular domain (NICD). In healthy cells, notch signaling is highly controlled and kept dormant by a repressor complex. When Notch signaling is activated, NICD proceeds through nuclear translocation and replaces the other corepressors to form an NICD–CSL complex. Hes‐1, c‐Myc, HER2, NF‐B, cyclin‐D1, and p21 are Notch target genes that are activated by the NICD–CSL complex through the recruitment of MAML and p300 coactivators.^56^, ^57^, ^87^ Notch signaling has a variety of functions during normal cell development, including the formation of the heart, liver, pancreas, central nervous system, and vasculature. Additionally, it controls adult progenitor stem cell functions in the skeletal muscle system, hematological system, skin, and gut.^88^, ^89^

By controlling CSC traits, Notch signaling hyperactivation helps tumor cells survive in challenging microenvironments. The involvement of Notch signaling in controlling CSCs of hepatocellular carcinoma, glioblastoma, breast cancer, and pancreatic cancer has been documented in a number of studies.^90^, ^91^, ^92^, ^93^ Notch signaling boosts breast cancer cell chemo‐resistance in low oxygen. HIF‐2 induction elevates NICD, Hey2, c‐Myc, Oct4, and NANOG. Notch inhibitor L685,458 lowers c‐Myc, NANOG, Oct4, indicating Notch's role in CSCs regulation. L685,458 also counters HIF‐2‐induced upregulation of CSC genes, mitigating stemness and resistance to paclitaxel in breast cancer.^91^ Hypoxia also controls GSCs by triggering the Notch 1 and Oct3/4 signaling pathways. When compared with a normoxic environment, Notch1, Oct3/4, and the cell surface SC marker CD133 were all significantly higher in glioblastoma cancer.^94^ Furthermore, Notch signaling has been demonstrated to control the GSCs’ diverse metabolic profile.^95^ Targeting the Notch system may help cancer patients recover more quickly from their treatments because Notch signaling has such a strong impact on various elements of cancer development and CSCs.

#### 3.1.4 Hedgehog signaling

Hedgehog signaling promotes morphogenesis and cell proliferation and, like other developmental pathways like Notch and Wnt signaling, is conserved from evolutionary point. Sonic hedgehog (Shh) pathway regulates cell development and cancer. Shh binds Patched‐1 (PTCH‐1), releasing Smoothened (SMO), facilitating Gli‐1 nuclear translocation, crucial for canonical and noncanonical signaling. The transcription of Gli‐1′s target genes c‐Myc, SOX‐2, NANOG, Oct‐4 and is then controlled by acting as a transcriptional activator.^33^ Early mammary gland development has been shown to be regulated by hedgehog signaling.^57^ Hedgehog signaling, however, has been shown to be aberrantly activated in a number of malignancies, including pancreatic, glioblastoma, chronic myeloid leukemia, multiple myeloma, and colon cancer.^96^

Hedgehog signaling has been demonstrated to control the formation of glioblastoma tumors and to support CD133+ GSC renewal. Gli1, Shh, and PTCH1 have been found to be overexpressed in GSCs.^97^ In our published investigation, we demonstrated that the antipsychotic drug penfluridol reduces the CSC markers Oct‐4, SOX2, and NANOG in glioblastoma by preventing noncanonical Akt‐mediated Gli‐1 inhibition.^98^ Multiple myeloma progenitor cells exhibit elevated SMO, akin to constant Gli‐1 activity. Inhibiting Shh signaling diminishes cancer cell stemness. Cyclopamine decreases GSC clonogenicity, self‐renewal, and CSC marker expression.^99^ SMO deletion causes the CML‐CSC population to decrease in the mouse model of CML.^100^ Hedgehog signaling has a crucial role in controlling CSCs; hence, inhibiting Hh signaling will be a successful cancer treatment strategy.

**FIGURE 3:**
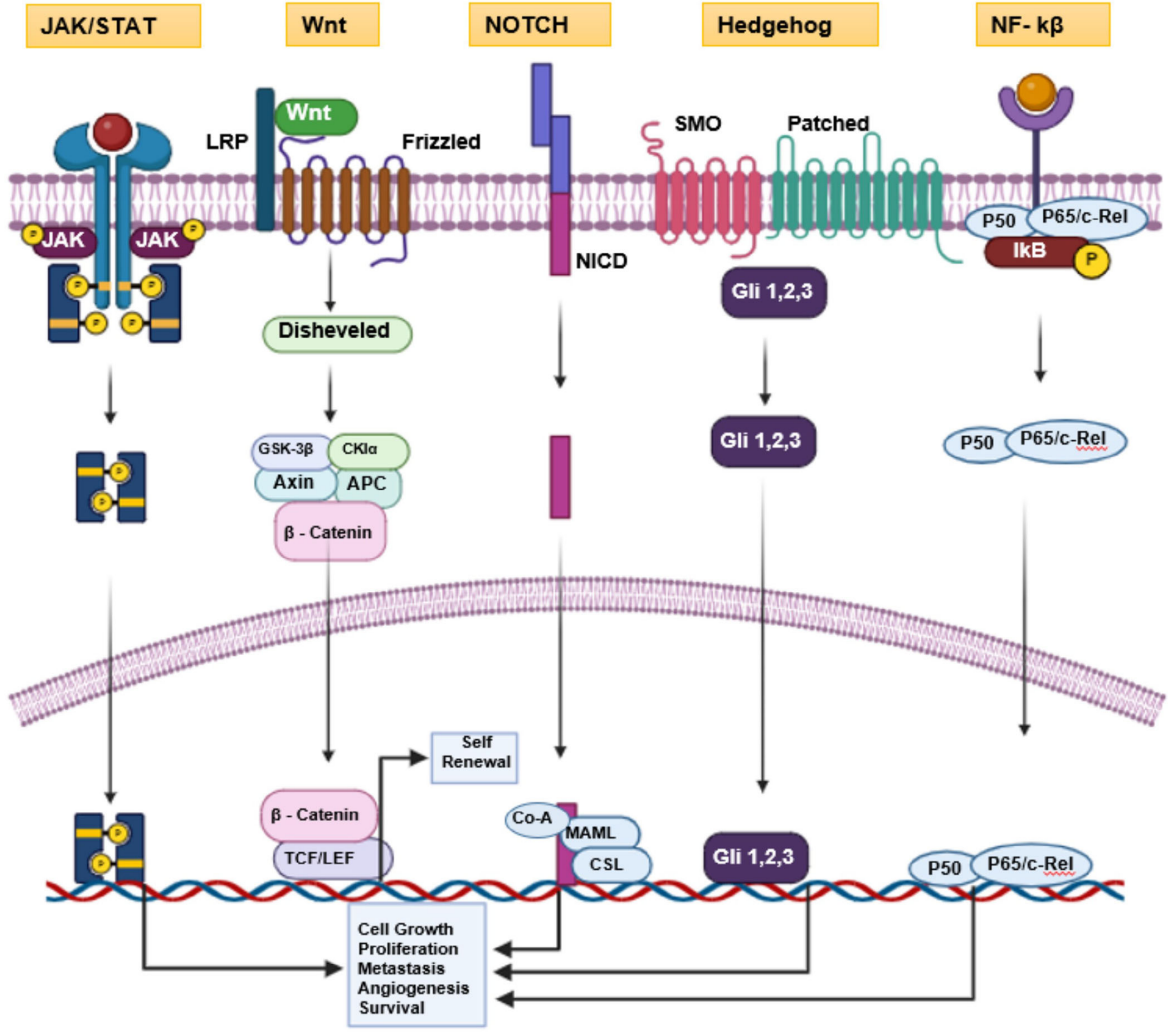
Demonstration of most widely researched cancer stem cell pathway and their functional impact of survival, invasion, and metastasis. Figure has been drawn with the help of Biorender.

The dysregulation of JAK/STAT, Wnt, Notch, and Hedgehog signaling pathways profoundly impacts the behavior of CSCs across multiple cancer types (Figure 3). Targeting these pathways holds significant therapeutic potential in combating cancer progression and overcoming treatment resistance. Further research into the molecular mechanisms underlying these signaling pathways and their crosstalk with CSC regulatory networks is essential for the development of effective and precise anticancer therapies, highlighting the importance of unraveling the complexities of CSC biology in oncology.

### 3.2 Endothelial–mesenchymal transition

EMT is a fundamental process in cancer progression, driving phenotypic changes associated with increased invasiveness, encompasses the augmentation of fibroid shape, resistance to cell‐death, and augmented ECM components.^101^ Recent studies highlight its pivotal role in cancer initiation, growth, and metastasis, particularly through its association with CSCs. The intricate network of signals from the TME, including CAFs, stromal cells, and growth factors, orchestrates EMT induction, mediated by transcription factors and microRNAs. Understanding this complex interplay is crucial for unraveling the mechanisms driving cancer metastasis and therapy resistance.

**FIGURE 4:**
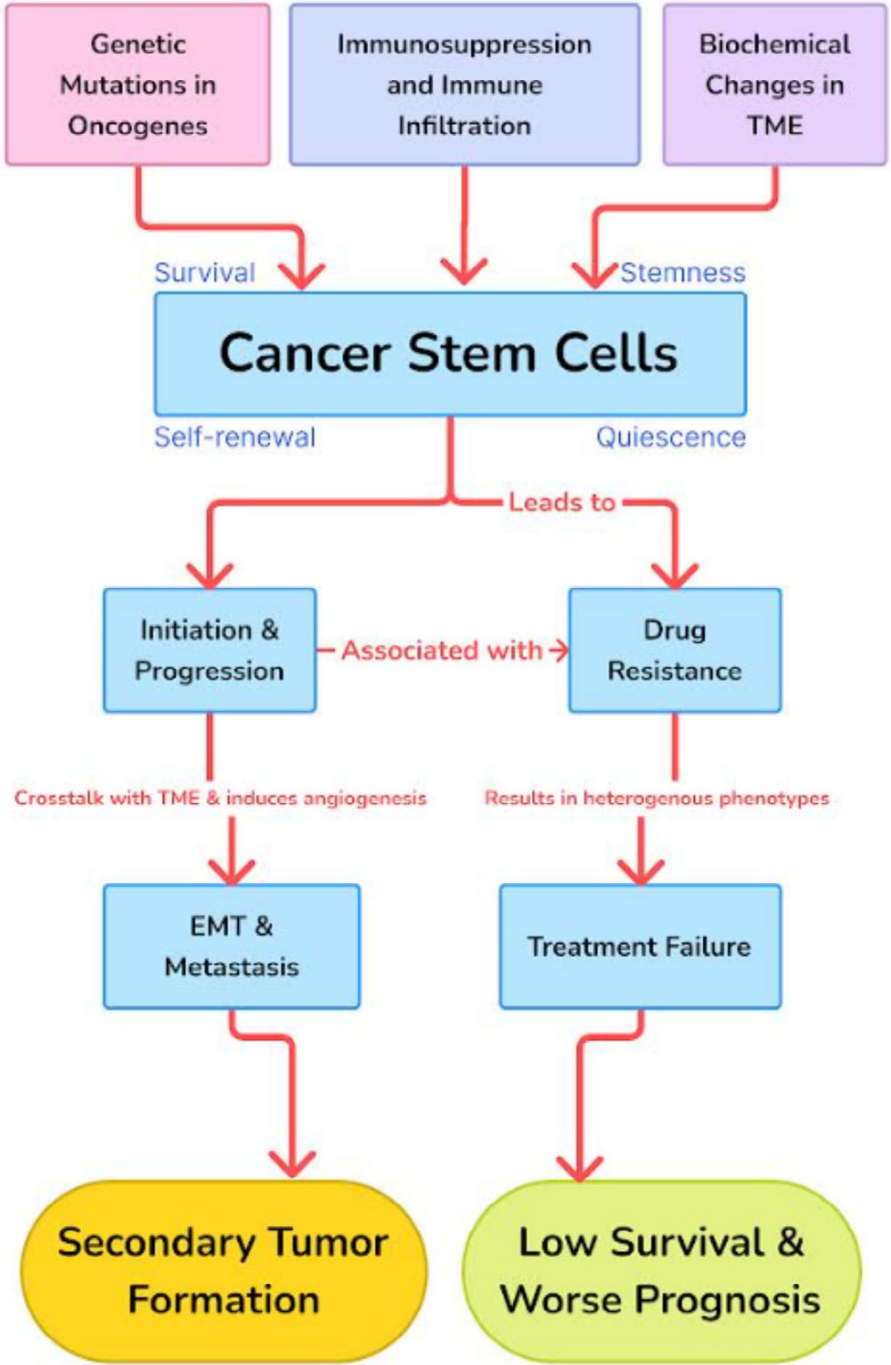
Schematic representation of the relationship between cancer stem cells, epithelial‐to‐mesenchymal transition, and metastasis. This figure illustrates the interconnected pathways and mechanisms linking cancer stem cells (CSCs), epithelial‐to‐mesenchymal transition (EMT), and the metastatic process in cancer progression.

The initiation of EMT inside the epithelial tissue of an organ is hypothesized to be among the primary stages in the development of cancer.^102^ The association between EMT and cancer growth, as well as the enhanced stemness of tumors, has been established in recent studies.^103^ The observed correlation between EMT and CSCs implies a possible direct link between CSCs and the ability of cancer to spread to other parts of the body (Figure 4). The correlation between elevated levels of EMT markers and the aggressiveness of metastatic illness has been shown, potentially due to the enhancement of stemness in tumor‐initiating CSCs and the inherent resistance to conventional treatments. Table 1 list biomarkers related to the cancer stemness, drug resistance, and metastasis.

**TABLE 1:** List of key biomarkers associated with cancer stemness, metastasis, and drug resistance, which are critical in understanding cancer progression and developing targeted therapies.

Category	Biomarker	Function/significance	References
Cancer stemness	CD44	Cell surface glycoprotein involved in cell adhesion and migration	^104^, ^105^
	CD133 (prominin‐1)	Cell surface protein often used as a marker to identify CSCs in various cancers	^106^, ^107^
	ALDH1 (aldehyde dehydrogenase 1)	Enzyme involved in oxidation of aldehydes; high activity is a marker of CSCs.	^108^, ^109^
	OCT4 (octamer‐binding transcription factor 4)	Transcription factor critical for maintaining stemness and self‐renewal	^110^, ^111^, ^112^
	SOX2 (sex determining region Y‐box 2)	Transcription factor that plays a role in maintaining pluripotency and self‐renewal	^113^, ^114^
	NANOG (Nanog homeobox)	Homeobox protein that is key in maintaining the undifferentiated state of CSCs	^115^, ^116^
Metastasis	E‐cadherin	Cell–cell adhesion molecule; its loss is associated with EMT and increased metastatic potential.	^117^, ^118^
	N‐cadherin	Cell–cell adhesion molecule; its expression is associated with EMT and enhanced migratory capacity.	^119^, ^120^, ^121^
	Vimentin	Intermediate filament protein; its upregulation is a hallmark of EMT and metastatic potential.	^122^, ^123^, ^124^
	SNAIL	Transcription factor that promotes EMT by repressing E‐cadherin expression	^125^, ^126^, ^127^
	SLUG	Transcription factor that promotes EMT and is associated with increased invasiveness.	^128^, ^129^
	TWIST	Basic helix–loop–helix TF that promotes metastasis via induction of EMT	^130^, ^131^
	MMPs (matrix metalloproteinases)	Enzymes that degrade extracellular matrix components, facilitating invasion, and metastasis	^132^
Drug resistance	ABCG2 (BCRP) (breast cancer resistance protein)	ATP‐binding cassette transporter involved in efflux of drugs from cancer cells, contributing to multidrug resistance.	^133^, ^134^
	ABCB1 (P‐glycoprotein)	ATP‐binding cassette transporter that pumps chemotherapy drugs out of cells, contributing to drug resistance	^135^, ^136^
	ALDH1 (aldehyde dehydrogenase 1)	Besides being a marker of CSCs, high ALDH1 activity is also associated with resistance to chemotherapeutic agents.	^137^, ^138^
	GLI1 (glioma‐associated oncogene homolog 1)	Transcription factor in the Hedgehog signaling pathway; its activation is linked to CSC maintenance and drug resistance.	^139^, ^140^
	NOTCH1 (neurogenic locus notch homolog protein 1)	Receptor in the Notch signaling pathway; its activation contributes to CSC maintenance and resistance to therapy.	^141^, ^142^
	BCL‐2 (B‐cell lymphoma)	Antiapoptotic protein that confers resistance to apoptosis‐inducing chemotherapy agents.	^143^, ^144^
	MCL‐1 (myeloid cell leukemia sequence 1 protein)	Antiapoptotic member of the Bcl‐2 family; contributes to survival and drug resistance in cancer cells	^145^, ^146^
	YAP/TAZ (Yes‐associated protein)	Transcription coactivators in the Hippo signaling pathway; their activation is associated with increased cell survival, stemness, and resistance to therapy.	^147^, ^148^
	TGF‐β (tumor growth factor‐ beta)	Cytokine that promotes EMT, CSC traits, and resistance to chemotherapy	^149^, ^150^

Functions and significance of each class of biomarkers in cancer initiation, progression, EMT, metastasis, drug resistance, signaling, and others have also been mentioned in the table. CD denotes cluster of differentiation markers represented with their respective CD numbers.

EMT can be induced by signals arising from the stroma that is associated with malignancies. Various types of stromal cells that are attracted to the blood vessels surrounding the tumor are connected to cancer cells.^151^ The EMT/MET programs are activated by a complex network of connections that are established by various stimuli in the TME.^152^ These signals include hepatocyte growth factor, platelet‐derived growth factor, and transforming growth factor‐beta (TGF‐β). Growth hormones, factors, and cytokines are examples of paracrine and juxtacrine signals in such a microenvironment.^153^ Additionally, a set of transcription factors known as EMT‐inducing transcription factors, namely Snail, Slug, zinc finger E‐box binding homeobox 1 (ZEB1), ZEB2, Twist, Goosecoid, and FOXC2, play a crucial role in inducing EMT.^102^, ^154^, ^155^ The regulation of EMT may be attributed to a negative feedback mechanism that involves ZEB1, ZEB2, and TGF‐β. The significance of the TGF‐β/SMAD1/LEF/platelet‐derived growth factor axis in the context of EMT signaling has also been documented.^156^ In addition to transcription factors, microRNAs (miRNAs) also contribute to the process of EMT. More specifically, the downregulation of the miR‐200 family leads to the initiation of EMT. The current work is focused on elucidating the intricate interplay between these several elements.

The regulation of EMT and CSC processes occurs at the genetic level through many oncogenic pathways, including MAPK/ERK, TGFβ‐SMAD, JAK/STAT, PI3K‐AKT‐NFκB, and WNT/β‐catenin pathways.^157^ Besides this, the expression of EMT‐TFs is boosted by ROS, hypoxia, and morphogenic signaling pathways such as NOTCH and WNT. These signals work in concert to cause cancer cells to take on genotypic and phenotypic traits that are characteristic of either an epithelial or mesenchymal type.^158^ With disruption of cell adhesion, disruption of polarity, cytoskeleton changes, expression of MMPs (MMP‐1, 2, 9, 12, and 13), and degradation of the ECM that allows tissue dissemination. EMT in cancer development takes the same pattern as EMT.^159^, ^160^, ^161^ Notably, both stroma and cancerous cells are impacted by elevated MMP levels in the TME. MMP‐7 and MMP‐14 production is increased in stromal cells, which accelerates the breakdown of the ECM and encourages tumor invasion.^162^ Additionally, E‐cadherin can be cleaved by proteases using MMPs, which results in extracellular E‐cadherin fragments that promote motility.^163^ It is significant to note that expression of various MMP types is associated with a worse prognosis in a number of malignancies, including ovarian,^164^ breast,^165^ gastric,^166^ and colorectal cancers.^120^

EMT is a fleeting mechanism that only affects a small group of cells that are at the verge of invasion.^167^ However, hybrid E/M cells have been seen in a variety of malignancies, such as lung, ovarian, and breast cancers,^168^, ^169^ as well as in various tumor mouse models.^170^, ^171^ Therefore, in comparison with hybrid E/M cells that experienced a EMT to a lesser extent, circulating tumor cells (CTCs) in a completely mesenchymal condition exhibit lesser metastasis.^172^ CTC clusters, which are collections of a few to many cancerous cells joined together, float freely in the blood stream, and display a varied expression of EMT and epithelial biomarkers.^172^ Additionally, CTC clusters have a strong metastatic potential that takes benefit of both mesenchymal characteristics that support cell invasion and motility and epithelial characteristics that contribute to extravasation and colonization propensity.^173^, ^174^ It is noteworthy that it was recently shown that breast CSCs with a hybrid E/M state, identified as CD24+ CD44+ ALDH+, exhibited the highest degree of invasiveness.^175^, ^176^ These findings strongly imply that stemness features can be acquired and maintained by cancer cells through increasing cellular plasticity, which is reflected in the preserving a transitory epithelial–mesenchymal phenotype. Many recent studies demonstrated that in addition to EMT, some other oncogenic pathways, for instance the MET pathway, can also contribute to stemness features and elevate metastatic ability in cancerous cells, lending weight to this concept. For instance, it has been observed that downregulating EMT‐TFs enhances the expression of stemness factors and enhances the growth of prostate and bladder cancer cells as spheroids,^177^ which is the normal pattern of stem cell growth.^178^ Similar to this, inhibiting PRRX1, a transcription factor that triggers EMT, encourages breast cancer cells to acquire stem cell characteristics, improving their ability for self‐renewal and promoting development in mammospheres.^179^ The htert/zeb1 complex also plays a direct role in the regulation of e‐cadherin, hence facilitating the process of EMT in colorectal cancer.

Finally, EMT orchestrates a spectrum of changes in cancer cells, enhancing metastasis, chemoresistance, and stemness. Stromal signals and oxidative stress trigger EMT, promoting cellular plasticity and acquisition of mesenchymal traits. MMP‐mediated ECM degradation facilitates tumor invasion, while EMT‐driven chemoresistance and immune evasion pose therapeutic challenges. Notably, hybrid E/M cells and circulating tumor cell clusters exhibit diverse mesenchymal and epithelial features, highlighting the dynamic nature of EMT in cancer metastasis. Targeting EMT pathways offers a hope for tackling therapeutic drug‐resistance and improving patient responses to cancer therapies.

## 4 CANCER METASTASIS: CELLULAR PLASTICITY AND CSCs

EMT stands as a critical process in cancer biology, linking TME signals to cellular phenotypic changes associated with aggressive tumor behavior such as tumor heterogeneity and metastasis. The involvement of EMT in promoting cancer stemness and metastasis underscores its significance in cancer progression. Unraveling the regulatory mechanisms governing EMT, from stromal signals to transcriptional and microRNA‐mediated control, offers avenues for targeted therapeutic interventions aimed at impeding cancer metastasis and enhancing treatment efficacy. Continued research into the multifaceted nature of EMT promises to illuminate novel therapeutic strategies for combating cancer progression and improving patient outcomes.

The examination of primary and metastatic cancers in humans has revealed significant levels of heterogeneity in terms of genomics, phenotypes, and antigens.^180^ This heterogeneity plays a vital function in the failure of therapy and the advancement of the illness. The aforementioned issue presents a complex and demanding clinical and technological obstacle.^180^ Several mechanisms have been suggested to elucidate the phenomenon of intratumour heterogeneity. These include genomic instability, as proposed by McGranahan and Swanton,^181^ and hierarchical organization resulting from the presence of CSCs, as discussed by Kreso and Dick.^182^ The influence of selective pressure exerted by the immune system is likely to affect the antigen heterogeneity within the tumor.^183^ Cancer immune editing facilitates the elimination of highly immunogenic cancer cells by the immune system, hence fostering the growth of clonal tumors and subsequently reducing heterogeneity. On the other hand, the absence of immune selection is anticipated to result in an augmented diversity of neoantigens. A recent study conducted by McGranahan and coauthors^184^ examined the heterogeneity of neoantigens in tumor samples obtained from patients with lung cancer and melanoma. The findings revealed that patients with clonal tumors, which accounted for approximately 78% of the observed clonality, exhibited a higher vulnerability to T‐cell attack and displayed greater sensitivity to tumor checkpoint inhibition in comparison with tumors with higher levels of heterogeneity, which constituted approximately 8% of the clonality. Additionally, McGranahan and coauthors^184^ also revealed varying numbers of cytotoxic CD8+ T cells in distinct locations of heterogeneous tumors. Inactivating the DNA repair machinery in colorectal, breast, and pancreatic cell lines results in an increase in the number and diversity of neoantigens in vivo, as well as the development of new antitumor T‐cell repertoires.^185^, ^186^ It is noteworthy that there exists a positive correlation between tumors exhibiting a high burden of neoantigens and favorable prognosis in lung cancer patients who undergo anti‐PD1 treatment, as demonstrated by Rizvi and coauthors.^185^ The efficacy of contemporary immunotherapies is contingent upon the capacity of the immune system, specifically T cells, to identify and eradicate cancers that include multiclonal or subclonal neoantigens. The aforementioned findings underscore the significance of gaining a more comprehensive comprehension of the intricate interplay between cancer cells and immune response, as well as the role of these interactions in the development of cancer heterogeneity.

The replicative capacity of CSCs allows for higher tumor heterogeneity, and it has been argued that stemness promotes tumor clone diversity.^182^, ^187^ Based on the notion, stemness may potentially promote intratumoral heterogeneity by blocking the immune system from eliminating newly formed cancer cells. As far as we know, these hypotheses have not been thoroughly examined either within or across different types of cancers. Scientists utilized data from two recent TCGA studies to conduct a comparative analysis of stemness and intratumoral heterogeneity.^156^, ^188^ The median stemness and median number of clones in cancer were found to have a strikingly positive correlation using data from the first study (*n* = 935 patients across 11 malignancies; *p* = 0.75; *p* = 0.008.^156^ Additionally, in a linear model that controlled for the cancer site and tumor purity, we discovered a significant connection between stemness and tumor clone count, demonstrating that these associations are perceptible both across and within malignancies (*p* = 0.0002). In a separate analysis based on the TCGA dataset, researchers discovered a comparable and statistically significant correlation (*p* = 0.64; *p* = 0.002; *n* = 6791 samples across 21 malignancies) by utilizing pan‐cancer predictions of intratumoral heterogeneity. Furthermore, the significance persisted across all samples even after controlling for cancer site and tumor purity using a linear model (*p* < 10^15^).^188^

Research has illuminated significant heterogeneity within CSCs, unveiling a complex landscape in tumor biology. For instance, recent investigations into head and neck cancer have delineated distinct CSCs phenotypes within the same histopathological tumor type. This diversity extends beyond mere phenotypic disparities among tumor cells to encompass variations among CSCs populations. A study by Xiao et al.^189^ explored the heterogeneity of CSCs in head and neck squamous cell carcinoma, revealing distinct molecular profiles and functional characteristics among CSCs subpopulations.^190^ Similarly, in a recent investigation by Dirkse et al., intratumoral heterogeneity in glioblastoma stem cells was unveiled, demonstrating the existence of multiple CSCs subsets with diverse gene expression patterns and tumorigenic potential. These findings underscore the dynamic nature of CSCs populations within tumors and emphasize the imperative for further research to elucidate the underlying mechanisms driving this heterogeneity.

Cancer metastasis has also been connected to other elements of cancer biology, such as chemoresistance and the prevention of cellular senescence.^93^, ^191^, ^192^ The ZEB1/2 case is an intriguing one. The cyclin kinase inhibitors p15INK4B, p16INK4A, and p21 are suppressed by these TGF‐induced EMT‐TFs, which eliminate EGFR‐dependent senescence in esophageal cancer.^193^ Similar to Ras signaling, TWIST disabling p53‐ and Rb‐dependent pathways prevents oncogene‐induced cellular senescence.^194^ Finally, EMT‐TFs' effects on cell‐survival pathways, primarily MEK/ERK and PI3K/AKT, as well as apoptosis associated genes, including members of the Bcl2 family, that impart lower sensitivity to apoptosis during EMT.^195^, ^196^

The chemoresistance to cytotoxic CD8+ T‐lymphocytes is associated to EMT and CSCs.^197^ Chemoresistance to chemotherapy has been linked to the activation of EMT in various tumor types. Breast, non‐small lung, and colorectal cancer patients receiving chemotherapy showed an enrichment of cells expressing mesenchymal biomarkers.^198^, ^199^ These findings are supported by the discovery that EMT‐TF inhibition and regulation of EMT (at posttranscription levels) repeal EMT‐induced drug‐resistance in lung and pancreatic carcinomas, respectively.^94^, ^192^, ^200^ Chemoresistance may be brought on by the systematic and sequential triggering of the several biochemical processes involved in EMT that consequently render cancer cells acquiring stemness characteristics. Cancer cells with high levels of the EMT‐TFs ZEB1, SNAIL1, and SNAIL2 express stemness factors SOX2, BMI1, and OCT4.^201^, ^202^, ^203^, ^204^ Notably, the CSCs fraction inside the tumoral mass is known to be characterized by mesenchymal and stemness features, which are in charge of tumor dissemination and nonresponsive to conventional therapy.^204^ Thus, throughout organogenesis, EMT may convert terminally differentiated epithelial cells to cells with more malleable, EMT and stem‐like characteristics as of pluripotent embryonic cells.

## 5 DRUG RESISTANCE MECHANISMS IN CSCs: REVISITING THE CSC THEORY

As discussed earlier, ECM is a network of biochemical molecules made up of glycoproteins, proteoglycans, and polysaccharides, supports CSC structurally, and is essential for the development of cancer.^205^ Through its interactions with a number of growth factors that interact with the membrane proteins of CSCs, the ECM creates an ideal milieu for the maintenance and growth of CSCs.^206^ By shielding the CSCs from chemotherapeutic drugs, ECM also contributes to treatment resistance.^207^ Hyaluronan synthase, another ECM component, is said to promote breast CSCs development.^208^ Laminin alpha 2, another element of the ECM, promotes self‐renewal and cell differentiation in glioblastoma stem cells.^209^ Increased levels of hyaluronan in the ECM interact with Oct4–Sox2–Nanog in head neck cancer‐related CSCs to enhance clonal formation and drug resistance.^210^ These results suggest that the ECM constituent laminin and hyaluronan, both contribute to the initiation and progression of cancer cells and subsequently resistance to treatment. This interaction underscores TME's influence on drug resistance and tumor advancement, highlighting the therapeutic potential of targeting TME components to combat resistance and enhance clinical outcomes.

The CSCs theory underscores the pivotal role of distinct stem cell populations in driving tumor proliferation, characterized by cellular heterogeneity, self‐renewal, and treatment resistance. Multidrug resistance (MDR), attributed to CSCs, involves mechanisms such as endogenous detoxifying enzymes, drug efflux, and DNA repair activity. CSCs also influence intratumoral heterogeneity, impacting therapy efficacy and immune response. Recent analyses using TCGA data reveal correlations between stemness and tumor clone diversity across various malignancies. This heterogeneity, governed by genomic instability and immune selection, poses significant challenges in cancer treatment, emphasizing the need for an in‐depth understanding of the intricate dynamics between CSCs, tumor heterogeneity, and immune response.

According to the CSCs theory, the proliferation of tumors is sustained by distinct populations of stem cells. The model in question is derived from four fundamental characteristics, namely cellular heterogeneity, self‐renewal, restricted plasticity within the tumor hierarchy, and treatment resistance.^211^ The issue of MDR in cancer treatment is mostly attributed to the presence of CSCs, which are generated by the expression of endogenous detoxifying enzymes, elevated levels of drug efflux, reduced drug response, hypoxic stress within the TME, and heightened DNA repair activity.^212^, ^213^, ^214^, ^215^, ^216^ The resistance to CSCs drugs is mediated through the activation of stem cell signaling pathways. The expression of ATP‐binding cassette (ABC) transporters is seen, which are known for their ability to confer MDR and mitigate the risk of drug‐induced harm. Despite the occurrence of cellular injury, specific CSCs indicators, such as stem pathways, play a role in mitigating oxidative stress by eliminating free radicals and promoting resistance to chemotherapeutic treatments. CSCs are also responsible for the activation of DNA repair mechanisms in tumor cells, hence playing a role in safeguarding against apoptotic agents.^116^, ^211^

The CSCs theory provides valuable insights into tumor progression and therapy resistance, attributing MDR to CSCs’ unique characteristics and signaling pathways.^20^ Stemness fosters tumor heterogeneity and resistance mechanisms, influencing treatment outcomes. Moreover, the correlation between stemness and intratumoral heterogeneity highlights the complexity of tumor evolution and immune selection. Understanding these dynamics is crucial for developing effective therapeutic strategies and immunotherapies targeting heterogeneous cancer populations. Future research should focus on elucidating the intricate interplay between CSCs, tumor heterogeneity, and immune response to improve treatment efficacy and patient outcomes in cancer therapy.

In conclusion, the intricate interplay between CSCs and the TME underscores their pivotal role in tumor development and progression. The TME, comprising fibroblasts, immune cells, and various signaling molecules, intricately regulates CSCs self‐renewal and differentiation pathways, crucial for maintaining CSCs stemness. CAFs emerge as key orchestrators of TME dynamics, contributing significantly to tumor progression, invasion, and metastasis through the secretion of various factors. Targeting CAFs holds promise as a therapeutic strategy to disrupt CSC‐mediated processes and improve patient outcomes across multiple cancer types. Understanding these interactions offers new avenues for precision medicine approaches in cancer therapy.

## 6 RELATIONSHIP BETWEEN CSCs, DRUG EFFLUX, AND DRUG RESISTANCE

ABC transporters, crucial for exporting toxic substrates, contribute to drug resistance in cancer, particularly in CSCs. ABCG2 was initially linked to the side population (SP) phenotype, though its role remains debated. Various factors like hypoxia and DNA methyltransferase activity regulate ABCG2 and SP populations. However, SP heterogeneity complicates interpretation, with ABCG2 identifying fast‐cycling tumor progenitors while ABCG2‐negative cells possess primitive stem‐like characteristics. Other ABC transporters like ABCB5 and ABCB1 also contribute to CSC‐mediated chemoresistance. Despite targeted inhibition, MDR remains, demanding further understanding of SP‐related pathways and ABC transporter compensation.

ABC transporters have direct contribution in the acquisition of resistance, and CSCs exhibit increased ABC transporter expression.^193^, ^217^ ABCG2 was the first of this kind of transporters recognized to display the SP traits. The concept posits that the SP fraction, that behave differently than the main population, can be recognized as CSCs, hence supporting CSC‐mediated treatment resistance. There is a correlation between the level of expression of ABC transporters and the cells’ maturation state. Cells that have the highest export activity are the least developed.^218^ A more lucid study was undertaken in 2001 and the outcomes showed that ABCG2 was an indicator of the SP phenotype and could be a potential biomarker for CSCs.^219^ Many factors such as amino acids (glutamine), epigenetic enzymes (DNA methyltransferase) activity, and oxidative status (HIFs) can regulate the SP fraction by controlling ABCG2.^220^, ^221^ However, the functional implications of ABCG2 in the SP fraction is largely controversial. The ABCG2+ population did not display considerable chemoresistance compared with the ABCG2− population. Moreover, ABCG2− cells exhibited higher sphere formation than ABCG2+ cells.^222^, ^223^ Patrawala and coauthors^223^ demonstrated that ABCG2+ cancer cells can form ABCG2− cells, and ABCG2− cancer cells can also transform into ABCG2+ cells. Another study showed that ABCG2 activity was not accountable for the stemness phenotypes.^224^ Other subtypes of the ABC transporter family also play role in CSC‐mediated chemoresistance. ABCB5 was colabeled with CD133 and CD44 and clinically linked with chemoresistance.^225^, ^226^ Furthermore, ABCB5 controls chemoresistant, which is probably linked to cell cycle checkpoints.^227^, ^228^ ABCB1 is another important ABC transporter contributing to the chemoresistance‐phenotype of CSCs by PKC/PI3K/Akt.^229^, ^230^

**FIGURE 5:**
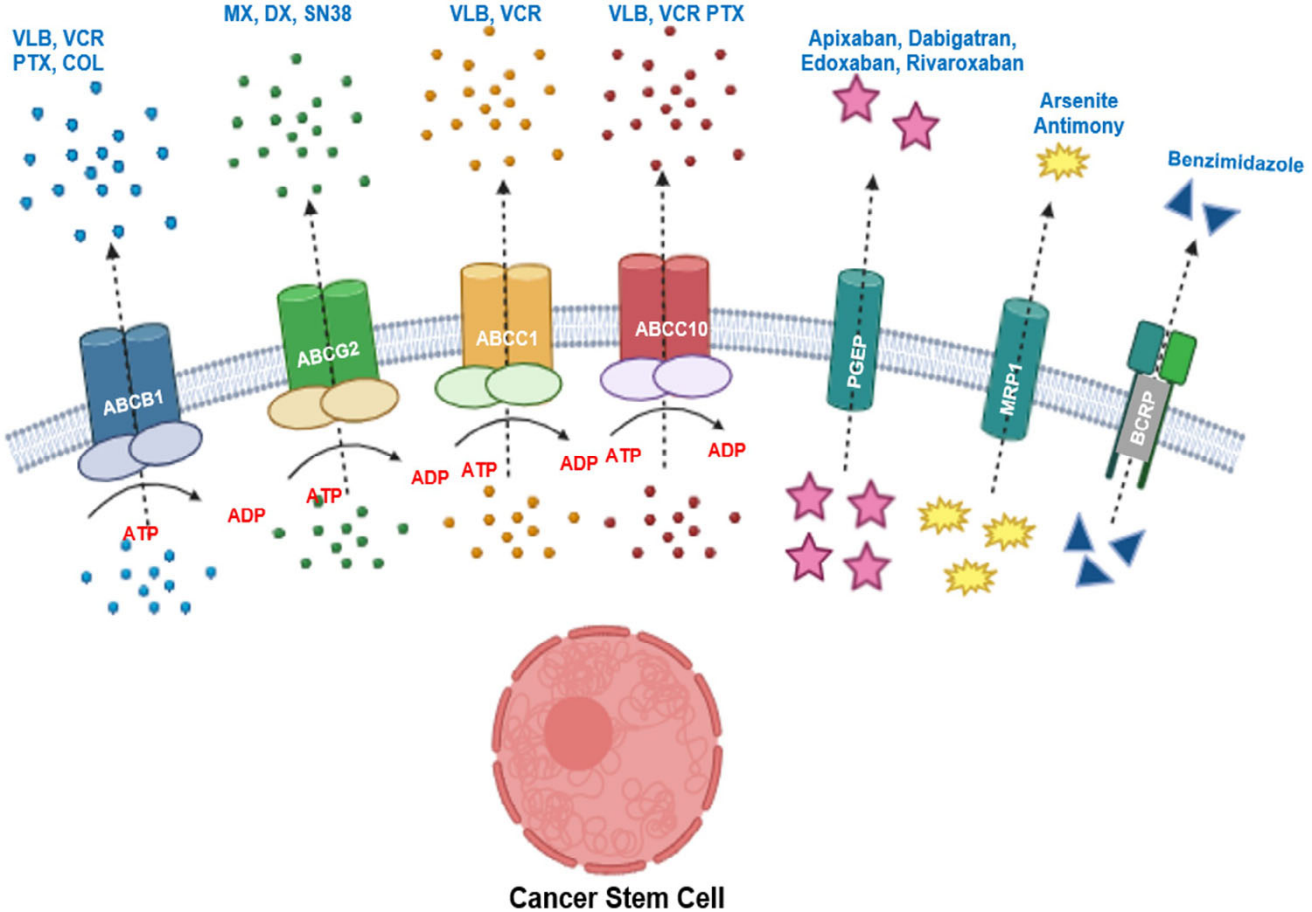
Mechanistic overview of drug efflux pathways implicated in drug resistance in cancer stem cells. Figure has been drawn with the help of Biorender.

ABC transporters, especially ABCG2, ABCB5, and ABCB1, regulate therapeutic resistance in cancer (Figure 5). Although specific ABC transporters are inhibited, cancer cells display an MDR phenotype. Tepotinib considerable alter ABCB1‐mediated MDR but not ABCC1‐ or ABCG2‐mediated MDR.^231^ While this phenotype can protect cells from cytotoxic agents, MDR genes are responsive to therapeutic agents such as doxorubicin and mitoxantrone.^232^, ^233^ One ABC transporter's blockage can be compensated for by the presence of numerous ABC transporters in CSCs. Uncertainty surrounds the precise mechanisms governing ABC transporter expression, which is essential to stem cell function. Clinical failures are primarily caused by a lack of study and knowledge regarding the SP percentage. However, the SP fraction's heterogeneity challenges interpretations, with ABCG2 identifying fast‐cycling tumor progenitors rather than exclusively CSCs. While specific inhibitors target ABC transporters, MDR persists due to compensatory mechanisms and a limited understanding of SP‐related pathways. Further research in this directed is needed to elucidate these pathways and improve clinical outcomes, highlighting the complexity of drug resistance mechanisms in CSCs and the necessity for developing comprehensive therapeutic strategies.

## 7 THERAPEUTIC TARGETING OF CANCER STEMNESS AND METASTASIS

Cancer is a substantial worldwide burden of disease. Historically, surgery, radiation, and chemotherapy constituted the primary modalities for cancer treatment. The theoretical foundation for understanding the initiation and progression of malignancies is provided by the concept of cancer stemness. CSCs were initially perceived as rare and inactive, establishing a one‐way structure within malignancies. They were believed to be responsible for the production of all cell fractions within a TME, occupying the topmost position in the tumor cell hierarchy. Hypoxia, mediated by HIFs, fosters CSCs proliferation, self‐renewal, and tumor formation while influencing EMT. The TME, comprising ECM components and ROS scavengers, further sustains CSCs characteristics and chemoresistance. Nevertheless, the constraints of effectiveness and the development of resistance to therapy remain unavoidable. The intricate interplay between hypoxia, CSCs, and the TME underscores their collective role in tumor progression and therapy resistance. The review commences by providing a concise overview of the historical findings, initial theories, and mechanisms that control CSCs, including WNT/β‐Catenin, PI3K/AKT, NF‐κB, JAK/STAT, hedgehog, Notch, TGF‐β, PPAR, and their interconnections. Targeting these elements offers promising avenues for therapeutic intervention to combat tumor growth and enhance treatment efficacy.

Subsequent investigations have revealed that the model of CSCs is complicate and subject to constant change. The field of cancer treatments is continuously evolving and progressing, with the advent of precision medicine and immunotherapy offering additional possibilities for patients to some degree. The majority of regular cells that display MHC class‐I are not recognized by NK cells. Nevertheless, tumor cells and CSCs that decrease the expression of MHC class‐I, while increasing the expression of activating ligands become the main focus of NK cell‐induced cell death.^234^, ^235^ However, the multifaceted nature of TME interactions presents challenges in developing effective therapeutic strategies, necessitating further research for innovative approaches to overcome therapy resistance in cancer.

Chimeric antigen receptor (CAR)‐T cancer therapy is also a promising therapeutic strategies that specifically targets CSCs.^236^ This technique has a distinct benefit compared with TILs therapy or ex vivo activation of autologous unaltered T cells since CSCs often show reduced ability to present antigens due to the downregulation of MHC and/or antigen‐processing and presentation molecules. CAR‐T cells can identify tumor cells independently of the MHC complex.^237^, ^238^ Zhu et al.’s^239^ research showed that CAR‐T cells specifically developed to target CD133 have the ability to efficiently eradicate CD133+ CSCs in glioma patients, whether in laboratory settings or under experimental conditions in model organisms. However, despite their effectiveness, these therapies have not been able to completely eliminate tumors, perhaps due to the terminally differentiated or aged CAR‐T cells. When glioma cells expressing CD57 (T cell senescence marker) interact with CAR‐T cells, there is a reported rise in the expression of the CD57 on CAR‐T cells.^239^ Likewise, CAR‐T cells that specifically target the epidermal growth factor receptor version III (EGFRvIII) have proven to be effective in eliminating the intended cells. This receptor has been recognized as a tumor‐specific antigen for GSCs.^240^ Nevertheless, during a phase I clinical trial conducted on individuals with glioblastoma, the administration of EGFRvIII‐targeted CAR‐T cells led to a considerable increase in the expression of inhibitory molecules and a decrease in tumor antigens.^241^ Further studies are required to enhance the effectiveness of CAR‐T cell treatment. Several CAR‐T treatments have been developed in recent years to specifically target antigens linked to CSCs, including CD22, CD123, and ALDH. Altogether, we envisage that studies on CAR‐T treatment will hold a significant focus in future research efforts, preclinical studies and clinical trials.

Furthermore, CIK cells have been shown to be capable of eliminating CSCs in preclinical models of different cancers, including melanoma, hepatic cancers, and others.^242^, ^243^ CIK cells elicit caspase‐3‐dependent programmed cell‐death and G2/M arrest in liver CSCs.^244^ CIK cells produce direct cellular toxic effects in cancers.^242^, ^243^ CIK cells are considered promising candidates for targeting CSCs due to their affordability as compared with other immune cells and their responsiveness to CSCs that are resistant to chemotherapy and precision therapies. These cells are easily derived from patients who have undergone these treatments.^245^ Consequently, the integration of CIK therapy with chemotherapy or precision therapies may serve as a potential future direction for immunotherapy.

Phenotypic plasticity is a characteristic of CSCs, which enables them to alter their characteristics in response to the microenvironment.^246^ This capacity leads to the development of malignancies that are genetically diverse. The growth of subpopulations that have enhanced capacities to renew themselves and resist therapy is facilitated by competitive interactions between various subclones within tumors. Research in the discipline was initially initiated by the application of classical cell sorting techniques. Nevertheless, our understanding of the CSC model has been substantially improved and refined as a result of the ongoing development of sequencing tools.^247^, ^248^ The development of cell sorting methods has transitioned from the use of physical attributes of cells, such as size, adhesiveness, cell‐density, and refractive properties, to the emphasis of specific cell surface markers, phenotypes and functionalities, such as dye efflux, calcium ion concentration, and pH. The study employs density gradient centrifugation, fluorescence‐activated cell sorting, magnetic‐activated cell sorting, and SP cell sorting.^20^

## 8 CLINICAL IMPLICATIONS AND CHALLENGES

Mounting data indicate that the population of CSCs, which is a subgroup of cancer cells, is accountable for the resistance to chemotherapy and the recurrence of cancer. CSCs has the ability to undergo self‐renewal and asymmetric cell division allow one progeny to maintain its stem cell features. Due to the absence of consensus on the criteria for identifying CSCs, it has not been possible to accurately determine the proportion of the CSC subpopulation in a specific tumor, the significance of CSCs in clinical outcomes, or the source of CSCs. Originally, it was thought that CSCs composed of just a small fraction of the total cell population in a solid tumor. However, recent suggestions propose that as many as 25% of cancer cells may exhibit characteristics similar to CSCs. CSCs play a crucial role in the development of tumors, EMT, metastasis, and the maintenance of tumor heterogeneity. This includes studying how treatments impact the characteristics of CSCs and the changes in associated molecules, as well as investigating the role of CSCs in drug resistance. These findings indicate that CSCs can be effectively targeted for anticancer therapies.

A series of focused clinical trials has been initiated by researchers who possess an in‐depth understanding of the critical functionalities of CSCs in chemoresistance. The objective of these trials is to investigate novel treatment approaches for CSCs. The California Institute for Regenerative Medicine has launched two clinical trials focusing on CSCs, one utilizing an antibody‐mediated immunotherapy that targets CD47 marker and the other targeting ROR1.^249^ Early clinical studies have assessed the effectiveness of a humanized anti‐CD47 antibody called magrolimab, also known as Hu5F9‐G4 or 5F9. Magrolimab has demonstrated good tolerability in human subjects. The effectiveness findings of four phase 1 trials using magrolimab alone or in combination with rituximab or azacytidine for AML, NHL, and solid malignancies showed significant outcomes. The second clinical trial utilizes anti‐ROR1, a receptor involved in Wnt5a signaling, which has been associated with the maintenance, self‐renewal, and spread of CSCs. The experiment also showed promising results for the Anti‐ROR1 antibody (for instance, cirmtuzumab), which discourages ROR1‐dependent Wnt5a signaling.

Clinical trials related to CSCs can be broadly classified into three or more groups/categories. The initial category, which is informed by the ChemoID assay, establishes the subsequent treatment protocols by utilizing biopsy samples from diseased individuals prior to the treatment to increase the concentration of SCs and assess their response to chemotherapeutic drugs. The probability of patient mortality in the group that was guided by the ChemoID assay was significantly reduced, as evidenced by the results of a phase III clinical study, NCT03632135.^98^ This implies that in future the ChemoID assay has the potential to become a standard therapeutic and diagnostic method, comparable to genetic screening/testing.

The second class is concerned with the design of precise inhibitors molecules that target the pathways through which CSCs cause therapeutic resistance. This class constitutes a diverse array of clinical trials, for instance those that utilize vismodegib or PF‐04449913 to obstruct the hedgehog pathway, LGK974 to target the WNT signaling, and OMP‐52M51 to counteract DLL4 in the Notch pathway. Despite the fact that γ‐secretase inhibitors are the most extensive category of medications that target the Notch pathway, the results of trials involving these medications are still undisclosed. Besides this, the progression of the medications RO4929097 and PF‐03084014 has been stopped for a variety of reasons.^250^, ^251^ Using human breast cancer graft, Schott et al.^21^ evaluated the effect of this inhibitor on the BCSC population, and the efficacy of a combination therapy, involving both MK‐0752 and DTX. The study showed that MK‐0752 targets the BCSC population. In parallel, a clinical trial (NCT00645333) was carried out in 30 patients with advanced BC, treated with increasing doses of MK‐0752 together with DTX.^21^ This work was aimed at determining the maximum tolerated dose of the inhibitor, administered sequentially with DTX, and to evaluate BCSC biomarkers in tumor biopsies. A decrease in CD44+/CD24−, and ALDH+ was seen in tumors from patients with this combination therapy. The study with the identifier NCT04137627 investigated the effect of melatonin as an antioxidant in conjunction with neoadjuvant therapy on the expression of tumor stem‐like features in oral squamous cell carcinoma.^252^ Nevertheless, the results did not indicate any substantial difference, despite a reduction in the expression of miR‐210 and CD44. This implies that the TME may not be the primary determining factor, despite the fact that it may contribute to the resistance mechanisms of CSCs. In theory, PARP inhibitors, which are involved in DNA repair, have the potential to be beneficial against CSCs. Nevertheless, the impact of these inhibitors on CSCs or biomarkers at pre‐ and posttreatment time has not been evaluated in the current clinical trials. Thus, additional research is required to investigate their impact on CSCs.

The third group concentrates on the treatment of CSCs by targeting specific markers, such as CD44v6, that are present. Bivatuzumab mertansine, an antibody–drug combination that specifically targets CD44v6, has been shown to enhance the precision of chemotherapy and has yielded convincing results in several cancer therapies.^253^ Nevertheless, the efficacy of the treatment against CSCs, as indicated by the study NCT02254005, remains ambiguous.

There may be some additional categories with varied approach for targeting CSCs, for instance vaccines and so on. Dendritic cell (DC) vaccines loaded with CSCs have progressed to the clinical application stage due to positive preclinical findings.^254^, ^255^ The first clinical trial of a DC vaccine loaded with CSCs in glioma patients was limited to only seven participants. However, the trial showed that these patients had a longer period of time without disease progression (progression‐free survival) compared with previous cases.^256^ The safety of DC vaccines targeting CSCs has been verified through the absence of significant adverse effects in further clinical trials involving patients with lung and pancreatic cancer. Additionally, researchers explored the potential clinical benefits of targeting CSCs in patients with refractory, progressed, or advanced tumors. Therapeutic treatments that specifically target CSCs, such as monoclonal antibodies, tyrosine kinase inhibitors, CAR‐T cells, and tumor vaccines, have been created and examined in clinical studies.^116^

To summarize, CSCs have an impact on the effectiveness of therapy, and it remains challenging to find the optimal treatment approach for targeting CSCs. Results from clinical studies indicate that the potential to reestablish chemotherapeutic resistance by targeting CSCs is currently uncertain. Although preclinical findings suggest that it is possible to target oncogenic pathways inside CSCs to eradicate them, there are few clinical trials that support this as of today. To this end, the prevailing concept at present is to elucidate the regulatory mechanisms and identify biomarkers of CSCs. The expected results of these clinical trials in the time ahead are avidly anticipated.

## 9 CONCLUSION

CSCs are dormant, pluripotent, self‐renewing neoplastic cells that were initially discovered in hematologic tumors and, thereafter, in solid tumors. This review summarizes the role of CSCs in tumor progression. The aggressiveness of the tumor is primarily governed by a subpopulation of CSCs, which commonly defy therapeutic procedures and have the ability to begin tumor growth even after the majority of tumor cells have been eliminated. CSCs are initiated through the activation of stemness‐related transcription factors, which are accountable for regulating the expression of genes that are not normally produced in normal cells but are highly expressed in CSCs. There exists a number of potential CSC biomarkers in different cancer types (Table 2). There are also signaling pathways associated with CSC biomarkers (Table 3). The link between CSC, EMT, and metastasis reveals a possible direct relationship between CSC and cancer‐spreading capabilities. In general, CSCs play a crucial role in the development of tumors and, by employing various methods, contribute to the resistance of therapies. In order to effectively sensitize CSCs, it is imperative to incorporate innovative therapeutic strategies. These strategies should involve the utilization of drug combinations that specifically target ABC transporters, DNA damage repair mechanisms, metastatic processes, autophagic inhibition, ferroptosis induction, as well as TMV disruption and immunotherapies. Many signaling pathways, including MAPK/ERK, TGF–SMAD, JAK/STAT, PI3K–AKT–NFB, and WNT/‐catenin, regulate EMT and CSC pathways at the genetic‐level. The degree of resistance observed may be indicative of the cumulative changes occurring in several molecular pathways, wherein proteins associated with resistance become dysregulated.

**TABLE 2:** A concise catalog of cancer stem cell biomarkers in different cancer types.

	Surface markers				
Cancers	CD marker	Other marker	Intracellular markers	Associated signaling and biological process/pathways, if any	References
Acute myeloid leukemia (AML)	CD33	CLL‐1 (chronic lymphocytic leukemia)	ALDH (aldehyde dehydrogenase)		^257^, ^258^, ^259^
	CD123	TIM3 (T‐cell immunoglobulin mucin‐3)	Nanog (Nanog homeobox)	Disease relapse	^260^, ^261^
			Oct‐3/4 (octamer binding transcription factor)		^262^
			Sox2 (sex determining region Y‐box 2)		^263^
Colorectal cancer	CD24	EpCAM (epithelial cell adhesion molecule)	ALDH	Cell adhesion Cadherin–catenin Wnt pathway	^264^, ^265^
	CD44	LGR5 (leucine‐rich repeat containing G protein‐coupled receptor)	Letm 1 (leucine zipper‐EF‐hand containing transmembrane protein 1)		^266^, ^267^
	CD133		Nanog	Self‐renewal	^268^, ^269^
	CD166		Oct‐3/4	Regulation of stemness	^270^
			Sall4 (Sal‐like protein 4)		^271^, ^272^
			Sox2	Regulation of stemness	^273^
Liver cancer	CD24	EpCAM	AFP (alpha‐fetoprotein)	Wnt/B‐catenin	^274^, ^275^
	CD44		Nanog		^276^, ^277^
	CD90		Notch (neurogenic locus notch homolog protein)		^278^
	CD133		Oct‐3/4		^269^, ^279^
Gastric cancer	CD24	CXCR4 (chemokine receptor type 4)	ALDH	SDF1 Pathway	^280^, ^281^
	CD44	EpCAM	Letm 1		^282^, ^283^
	CD90	LGR5	Musashi2 (Musashi RNA binding protein 2)		^284^, ^285^
	CD133	LINGO2 (leucine rich repeat and Ig domain containing 2)	Nanog		^286^, ^287^
			Oct‐3/4		^288^
			Sox2		^289^
Breast cancer	CD24	CXCR4	ALDH	Wnt/B‐catenin	^290^, ^291^
	CD29	EpCAM	BMI‐1 (B cell‐specific Moloney murine leukemia virus integration site)		^292^, ^293^
	CD44	LGR5	Nanog		^294^, ^295^
	CD49f	ProC‐R	Notch		^296^, ^297^
	CD61		Oct‐3/4		^298^, ^299^
	CD70		Sox2		^300^
	CD90				^301^, ^302^
	CD133				^303^, ^304^
Chronic myeloid leukemia (CML)	CD25	IL1RAP	FOXO (forkhead box transcription factor)	Wnt/B‐catenin	^305^, ^306^
	CD26			JAK/STAT	^307^, ^308^
	CD33			Hedgehog/Gli2/Smo	^307^, ^309^
	CD36				^310^
	CD117				^311^
	CD123				^312^
Lung cancer	CD44	EpCAM	ALDH	Tumor sphere formation	^313^
	CD87		Nanog		^314^, ^315^
	CD90		Oct‐3/4	Tumor sphere formation	^314^, ^316^
	CD133			Chemoresistance	^269^, ^317^
	CD166			Activation of leukocyte adhesion molecule	^318^

The catalog also mentions about surface biomarkers as well as intracellular biomarkers. Association and implication of these biomarkers in different cell signaling pathways have also been presented in the table. CD denotes cluster of differentiation markers represented with their respective CD numbers.

**TABLE 3:** A concise catalog of cancer stem cell biomarkers associated with different biological processes and pathways implicated in cancers.

Targets	Biomarkers	References
Apoptotic pathway	Caspases, cFLIP (cellular FLICE‐like inhibitory protein), death receptors, Bcl2 (B‐cell lymphoma), Chk1 (checkpoint kinase), ATM (ataxia‐telangiectasia mutated), ATR (ataxia telangiectasia and Rad3 related), Chk2, MOMP (myotonic dystrophy protein kinase)	^319^, ^320^
Long noncoding RNA	ROR (retinoic acid receptor‐related orphan receptor), HOTAIR (HOX transcript antisense RNA), H19, UCA1 (urothelial cancer associated), and ARSR (arsenic acid‐resistant)	^123^, ^321^
Circular RNA	CircCTIC1 (circular cancer/testis antigen), CircZKSCAN1 (circular ribonucleic zinc finger stalk‐and‐knuckle motif containing), CircGprc5a (circular genomic region of protein coupled receptor class C G‐protein coupled receptor), CircPRMT5 (protein arginine methyltransferase 5), Circ008913, CircZEB1 (zinc finger E‐box binding homeobox), CircPTN (circular antisense PTEN), mmu_circ_0000730, CircRNA_103809, Cir‐CCDC66, Circ‐NOTCH1	^322^, ^323^
MicroRNA	hsa‐miR‐9, hsa‐miR‐16, hsa‐miR‐17, hsa‐miR‐20, hsa‐miR‐34a/b, hsa‐miR‐107, hsa‐miR‐152, hsa‐miR‐122, hsa‐miR‐181, hsa‐miR‐196, hsa‐miR‐200c, hsa‐miR‐215, hsa‐miR‐218, hsa‐miR‐301, hsa‐miR‐320, hsa‐miR‐328, hsa‐miR‐451, hsa‐miR‐452	^324^, ^325^
Transcription factors	NF‐κB (nuclear factor kappa‐light‐chain‐enhancer of activated B cells), AP1 (activator protein), STAT3 (signal transducer and activator of transcription 3), HIF‐1 (hypoxia‐inducible factor), MYC (myelocytomatosis oncogene), ETS1 (ETS proto‐oncogene 1, transcription factor), β‐catenin/TCF, Oct‐4, Sox‐2, Nanog	^113^, ^326^, ^327^
Drug resistance	FOXC2 (forkhead box C2), ABC (ATP‐binding cassette) transporters, PRRX1 (paired‐related homeobox), hypoxia, MRP (multidrug resistance‐associated protein), BCRP (breast cancer resistance protein), ROS (reactive oxygen species), EMT (epithelial–mesenchymal transition), P‐gp, BCR (breakpoint cluster region), ABL (Abelson murine leukemia viral oncogene homolog), ZEB1/2 (zinc finger E‐box binding homeobox), SNAIL, SLUG, TWIST1/2	^328^, ^329^, ^330^
Signaling pathways	Notch, JAK (Janus kinase 2), STAT (signal transducers and activators of transcription), PI3K (phosphoinositide 3‐kinase), Akt (Ak strain transforming), CSC markers LGR4−6, MAPK (mitogen‐activated protein kinase), mTOR, HIFs, R‐spondin 2, cytokines, Wnt, c‐Myc, c‐Met, AXIN2 (axis inhibition protein 2), MMP9 (matrix metalloproteinase‐9), MMP7, nestin, NANOG, β‐catenin, SOX2, Oct‐4, GSK‐3β (glycogen synthase kinase‐3beta), JAG (jagged canonical notch ligand), TACE (tumor necrosis factor‐α converting enzyme), ADAM17 (a disintegrin and metalloprotease), sonic hedgehog, PTCH1 (protein patched homolog 1), SMO (smoothened, frizzled class receptor)	^331^, ^332^, ^333^
Stem cell differentiation	CD133, CD90, BMPR1 (bone morphogenetic protein receptor), BMPR2, HNF4A (hepatocyte nuclear factor 4alpha), neuropilin‐1 receptor, HDAC (histone deacetylase)	^334^, ^335^
Tumor microenvironment	T‐regulatory cells, hypoxia, hyaluronan synthase, glycoproteins, proteoglycans, laminin alpha polysaccharides, NK (natural killer) cells	^336^, ^337^
Cell surface markers	MyD88, CD44, CD133, CD24, CD13, CD90, CD166, ALDH1A1, ESA	^338^, ^339^, ^340^

Biomarkers such as cell cycle check‐points, apoptosis biomarkers, transcription factors, receptors, transporters, immune cells, enzymes, glycoproteins, ECM proteins, physiological conditions have been mentioned alongside their respective target molecules, pathway or process.

Ultimately, the process of overcoming therapeutic resistance in CSCs is complex and necessitates the implementation of the following factors: (i) Enhanced comprehension of the mechanisms governing the resistance of CSCs to therapeutic interventions. (ii) Utilization of a combination of pharmacological approaches and structural modifications in drug design to address CSCs resistance. (iii) Integration of experimental techniques and molecular dynamic simulations to investigate the crystal structures, properties, and formation of specific proteins and agents targeted toward CSCs. (iv) Implementation of novel strategies such as targeting drug‐efflux pumps/transporters and the CSC microenvironment, inducing CSC apoptosis.

The majority of current clinical trials only employ signaling pathway inhibitors in patients. However, determining whether it is necessary to target CSCs for the antitumor impact is still difficult (NCT00106145, NCT01608867, NCT00844064).^20^ It is crucial to use more advanced clinical trial designs in order to determine the efficiency of targeting signaling pathways within CSCs.

State‐of‐the‐art cell sorting and high‐throughput next‐generation sequencing techniques such as single cell sequencing have allowed us to accurately detect changes in the genome or transcriptome, providing valuable information about the molecular changes and processes involved in cancer biology.^341^, ^342^ By employing single‐cell analysis, a higher level of detail in understanding the fundamental processes involved in cancer development is achieved. This approach allows for a comprehensive understanding that would otherwise be missed when sequencing entire samples, where the molecular characteristics of individual cells are combined and averaged across the entire cell population. In this article, we envisage the potential a brief summary of how single‐cell analysis is used in various aspects of CSC research. In addition, the capacity of current sorting and sequencing technologies to fulfill clinical diagnostic requirements and address the present obstacles are quite appreciable. Further research should be conducted to enhance preclinical models of CSCs, and there is a need to identify surrogate markers or functional assays that can effectively monitor the biological activity of CSCs and their responses to treatment.

## AUTHOR CONTRIBUTIONS

Tikam Chand Dakal conceptualized and prepared the outline of the review article. Tikam Chand Dakal, Ravi Bhushan, Caiming Xu, Bana Ram Gadi, Swaranjit Singh Cameotra, Vikas Yadav, Abhishek Kumar, Jarek Maciaczyk, and Ingo G. H. Schmidt‐Wolf, and Amit Sharma wrote the manuscript. Ravi Bhushan and Caiming Xu prepared figures and tables for the manuscript. Tikam Chand Dakal, Ravi Bhushan, Caiming Xu, Bana Ram Gadi, and Abhishek Kumar revised the manuscript. All authors read, reviewed, and approved the submitted version.

## CONFLICT OF INTEREST STATEMENT

All authors have showed no conflict of interest.

## ETHICS STATEMENT

Not applicable.

## Data Availability

Not applicable.
